# Transport of L-carnitine in human corneal and conjunctival epithelial cells

**Published:** 2010-09-04

**Authors:** Shunjiang Xu, Judith L. Flanagan, Peter A. Simmons, Joseph Vehige, Mark D. Willcox, Qian Garrett

**Affiliations:** 1 Brien Holden Vision Institute, The University of New South Wales, Sydney, NSW, Australia; 2 Allergan Inc., Irvine, CA; 3 School of Optometry and Vision Science, The University of New South Wales, Sydney, NSW, Australia

## Abstract

**Purpose:**

Previously we demonstrated expression and localization of carnitine/organic cation transporters, OCTN1 and OCTN2, in human corneal and conjunctival epithelia. The present study aimed to examine the characteristics of L-carnitine transporters in cultured human limbal corneal (HCLE) and conjunctival epithelial (HCjE) cells.

**Methods:**

Time-course, Na^+^-dependence, kinetics, energy- and pH- dependence of L-carnitine transport were investigated by monitoring L-[^3^H]carnitine uptake into HCLE and HCjE cells. To determine the specificity of action, competition and inhibition studies were performed.

**Results:**

The uptake of L-carnitine into HCLE and HCjE cells was saturable and time-dependent. An Eadie-Hofstee plot showed two distinct components: a high- and a low- affinity carnitine transport system in HCLE and/or HCjE cells. L-carnitine transport was significantly inhibited by the metabolic inhibitors (sodium azide, dinitrophenol, iodoacetic acid). The L-carnitine analogs (D-carnitine, acetyl-L-carnitine and γ-butyrobetaine), tetraethylammonium (TEA), 2-amino-2-norbornane carboxylic acid (BCH), strongly inhibited uptake of L-[^3^H]carnitine. Uptake of L-[^3^H]carnitine also required the presence of Na^+^ in the external medium and the uptake activity was maximal at pH 5.5. The anti-OCTN2 antibody blocked L-carnitine uptake in both HCLE and HCjE cells whereas the anti-OCTN1 antibody did not significantly block L-carnitine uptake.

**Conclusions:**

L-carnitine is transported into HCLE and HCjE cells by an active carrier mediated transport system that is time-, Na^+^-, energy- and pH- dependent. The carnitine/organic cation transporter OCTN2 appears to play a dominant role in this process.

## Introduction

Dry eye syndrome (DES) can result in epithelial desiccation and ocular surface irritation. These symptoms can greatly affect the quality of life for affected patients. One of the key factors in dry eye is an increase in tear osmolarity. This increase in osmolarity can adversely affect cells causing cell shrinkage and eventual death. To compensate for hypertonic conditions, several compatible solutes have been incorporated into topical formulations for the treatment and management of dry eye syndrome. These are organic compounds that work like electrolytes to balance osmotic pressure, yet do not interfere with cellular metabolism, thus aiding survival of organisms under extreme osmotic stress. L-carnitine is one such compatible solute, due to its documented osmoregulatory activities [1]. L-carnitine has been demonstrated as an osmoprotectant against hyperosmotic stress of corneal epithelial cells in vitro [2,3]. Further, the topical use of L-carnitine has been demonstrated to result in rapid and consistent improvements in the signs and symptoms of dry eye patients [4]. These observations suggest that L-carnitine may play a homeostatic role in the eye, in addition to its well known role in β-oxidation of fatty acids by facilitation of transport of long-chain fatty acids into the mitochondria as acylcarnitine esters [5,6]. This is consistent with the findings of others who have demonstrated lower carnitine levels in patients with dry eye syndrome than in healthy subjects [7]. Pescosolido and colleagues [7] speculated that an imbalance in the concentration of carnitine molecules in the tear film may be partially responsible for the damage to ocular cells exposed to the hypertonic tear film found in dry eye syndrome.

Topically applied L-carnitine is actively taken up by ocular cells in animal models [8,9]. Further evidence suggests the existence of a carrier-mediated organic cation transport process in the rabbit conjunctiva that mediates absorption of organic amines, although the underlying mechanisms have yet to be fully elucidated [8,9]. Previously, we have reported the presence of organic cation/carnitine transporters, OCTN1 and OCTN2, in human corneal and conjunctival epithelial cells, as well as rabbit corneal and conjunctival epithelium [10]. We have further demonstrated that OCTN1 and OCTN2 are predominately localized in the apical membrane of these cells [10]. However, the mechanism of facilitation of carnitine transport in corneal and conjunctival epithelium requires clarification.

Together with the organic cation and organic anion transporters (OCTs and OATs), the OCTN transporters (organic cation transporter novel type) belong to the SLC22A family within the solute carrier (SLC) superfamily [11]. The organic cation transporter (OCTN) subfamily comprises three members; OCTN1, OCTN2, and OCTN3 that transport the organic cations, L-carnitine, and acylcarnitines [12], differing in their affinity and capacity for compound transport, energization of transport, and sensitivity to inhibitors [11,13-16]. OCTN1 (SLC22A4) has been functionally demonstrated as a multispecific, bidirectional, and pH-dependent organic cation transporter, presumably energized by a proton antiport mechanism that transports L-carnitine in a Na^+^-dependent manner [17,18]. OCTN2 (SLC22A5) is unique in that it transports carnitine with high affinity in a Na^+^-dependent manner and transports organic cations in a Na^+^-independent manner [15,19]. The OCTN2 carnitine-specific transport system has been documented in human kidney, skeletal muscle, heart, and placenta [14,20]. OCTN3 (SLC22A21) meditates L-carnitine transport in a Na^+^-independent manner and has higher affinity for L-carnitine than OCTN1 or OCTN2 [17]. In addition, L-carnitine can also be transported by the CT2 (human carnitine transporter, SLC22A16) [21] and by ATB^o,+^ (amino acid transporter B^0,+^, SLC6A14) [22], which are Na^+^-independent and Na^+^-dependent transporters respectively. ATB^o,+^ is reported to be a low-affinity transporter for L-carnitine [22].

To further our previous investigation in which we demonstrated the expression of L-carnitine transport proteins in corneal and conjunctival epithelium [10], the present study extends the functional characterization of L-carnitine transporters through examination of the sodium-, energy- and pH-dependence, and substrate specificity of the transport process.

## Methods

### Materials

Cell culture media (keratinocyte serum-free medium, K-SFM), media supplements, and epidermal growth factor (EGF) were purchased from Invitrogen-Gibco (Grand Island, NY). L-carnitine, D-carnitine, acetyl-L-carnitine, tetraethylammonium (TEA), 2-amino-2-norbornane carboxylic acid (BCH), γ-butyrobetaine, sodium azide, dinitrophenol, iodoacetic acid, and Triton X-100 were from Sigma Chemical Co. (St Louis, MO). L-[methyl-^3^H]carnitine hydrochloride (specific activity of 3.07 TBq/mmol) was obtained from GE Healthcare UK Limited, Amersham Place, Little Chalfont (Buckinghamshire, UK). Goat anti-human OCTN1 polyclonal antibody and goat anti-human OCTN2 polyclonal antibody were from Santa Cruz Biotechnology, Inc. (Santa Cruz, CA). All other reagents were of analytical grade.

### Cell culture

Immortalized human corneal-limbal epithelial (HCLE) and human conjunctival epithelial (HCjE) cell lines derived from primary cultures of HCLE and HCjE cells (a kind gift from Ilene Gipson’s laboratory, Schepens Eye Research Institute, Boston, MA) were used. HCLE and HCjE cells were cultured as described previously [23,24]. Briefly, cells were maintained on plastic at 2×10^4^/cm^2^ in K-SFM, supplemented with 25 μg/ml bovine pituitary extract, 0.2 ng/ml EGF and 0.4 mM CaCl_2_ and were grown at 37 °C in a 5% carbon dioxide atmosphere. To enhance nutrient composition, the cultures were switched at approximately 50% confluence to a 1:1 mixture of K-SFM and low calcium DMEM/F12 (Invitrogen) to achieve confluence.

### Transport study

HCLE and HCjE cells were grown on 24-well tissue culture plates at an initial seeding density of 5×10^5^ cells/well to 80%–90% confluence. Following the removal of media, the cells were pre-incubated with uptake buffer (25 mM Tris/HEPES, 140 mM NaCl, 5.4 mM KCl, 1.8 mM CaCl_2_, 0.8 mM MgSO_4_, and 5 mM glucose, pH 7.4) at 37 °C in air for 60 min. L-[^3^H] carnitine (L-[methyl-^3^H]carnitine hydrochloride) was added to the medium in the presence or absence of unlabeled substrates of varying concentrations. Non-specific uptake for the labeled substrates was determined using 100 fold excess unlabeled L-carnitine (up to 10 mM due to solubility). To obtain the specific uptake, the nonspecific uptake values were subtracted from the total uptake values. At a given incubation time, the incubation medium was removed, and the cells were rinsed three times in ice-cold PBS for 30 s each. The cell membranes were then solubilized using 0.1 M NaOH and 0.1% Triton X-100, and aliquots were removed for liquid scintillation counting (disintegrations/min). The cellular protein content was measured using a LavaPep peptide quantification kit (Fluorotechnics Pty Limited, Sydney, Australia) with a BSA standard. For Na^+^-free experiments, NaCl was substituted with equimolar choline chloride in the above buffer [17].

The energy-dependence of L-[^3^H]carnitine uptake was investigated by pre-treating the cells with metabolic inhibitors, sodium azide (10 mM), dinitrophenol (10 mM), or iodoacetic acid (10 mM) for 30 min followed by incubation with 24 nM L-[^3^H]carnitine for 30 min.

To study the pH-dependence of the L-[^3^H]carnitine uptake, cells were pre-incubated for 60 min in the uptake buffer at pH 5.5, 6.5, 7.4, and 8.5, respectively. L-[^3^H]carnitine (24 nM) was then added and incubation was continued for 30 min.

Substrate specificity was examined by pre-incubating cells for 60 min in the uptake buffer followed by further incubation of cells for 30 min with L-[^3^H]carnitine (24 nM) in the absence (control) or presence of 0.5 and 1.0 mM of L-carnitine structural analogs (unlabeled L-carnitine, D-carnitine, acetyl-L-carnitine, or γ-butyrobetaine), or 0.1 and 1.0 mM tetraethylammonium (TEA, a known organic cation substrate for OCTN2) [25], and 0.1 and 1.0 mM 2-amino-2-norbornane carboxylic acid (BCH, a known specific inhibitor for ATB^0,+^) [22].

For blocking experiments, cells were pre-incubated for 60 min with uptake buffer in the absence (control) or presence of OCTN1 (1:500) and/or OCTN2 (1:500) antibody at 37 °C; L-[^3^H]carnitine (24 nM) was then added and the incubation was continued for 30 min.

### mRNA expression

mRNA expression was investigated as previously reported [10]. Briefly, total RNA was extracted from cultured HCLE and HCjE cells using the SV Total RNA Isolation System (Promega, Madison, WI). RT–PCR was performed using SuperScript One-step RT–PCR with Platinum Taq System (Invitrogen). Purity and integrity of RNA was verified using an ultraviolet spectrophotometer and agarose gel visualization of ribosomal bands, respectively. Transcripts for *ATB^0,+^* were amplified using the primers described in Table1. The transcripts for *OCTN1* or *OCTN2* were amplified as previously reported [10] using the primers listed in Table 1. A control housekeeping gene β-actin (*ACTB*) was amplified under the same conditions [10]. The PCR products were separated by electrophoresis on a 1.2% agarose gel and analyzed by Gel-Pro analyzer version 3.1 software (Media Cybernetics, Silver Spring, MD). The ratio of integrated density of target genes over *ACTB* was used to normalize the relative mRNA expression. The PCR products were purified using Wizard SV Gel and PCR Clean-up System (Promega). The identity of each PCR product was verified by DNA sequencing (Department of Biologic Sciences, Macquarie University DNA Analysis Facility, Sydney, Australia).

**Table 1:** Primer sequences.

Transporter	Primer sequence	T_M_ (°C)	Product size (bp)
*ATB^0,+^*	5’-TGTCTACCTCGGCCTCCTAA-3′	60	300
	5′-CCAAATCTTCCCTGAATTGC-3′	60	
*OCTN1*	5′-CTGGATGCTCCTAATTTACATGG-3′	49	785
	5’-AGGAGACTCTCTAGAAATGGTTGG-3′	49	
*OCTN2*	5′-AGTGGGCTATTTTGGGCTTT-3′	60	398
	5′-GGTCGTAGGCACCAAGGTAA-3′	60	

### Data analysis

The uptake experiments were routinely executed in duplicate and each experiment was repeated three to four times. The results are expressed as mean ±SD. The apparent kinetic parameters, *K*_m_ and the maximal transport rate (*V*_max_), of carnitine uptake by HCLE and HCjE cells were estimated by nonlinear regression curve fitting according to the following Michaelis–Menten type equation with two saturable transport components, where v and [s] are the velocity of substrate uptake and the substrate concentration respectively and indices 1 and 2 indicate the high- and low-affinity components respectively;

$$v=V_{\text{max1}}\;\times \;\left[s\right]/\left(K_{\text{m1}}+\left[s\right]\right)+V_{\text{max2}}\;\times \;\left[s\right]/\left(K_{\text{m2}}+\left[s\right]\right)$$

The *K*_m_ and *V* _max_ values were determined from Lineweaver-Burk plots. Student’s unpaired *t -*test was performed using commercial computer software (SPSS; SPSS Inc., Chicago, IL). Post hoc multiple comparisons were analyzed incorporating the Bonferroni correction. Statistical significance was set at p<0.05.

## Results

### Time course and Na^+^ dependence of L-[^3^H] carnitine uptake by HCLE and HCjE cells

**Figure 1:**
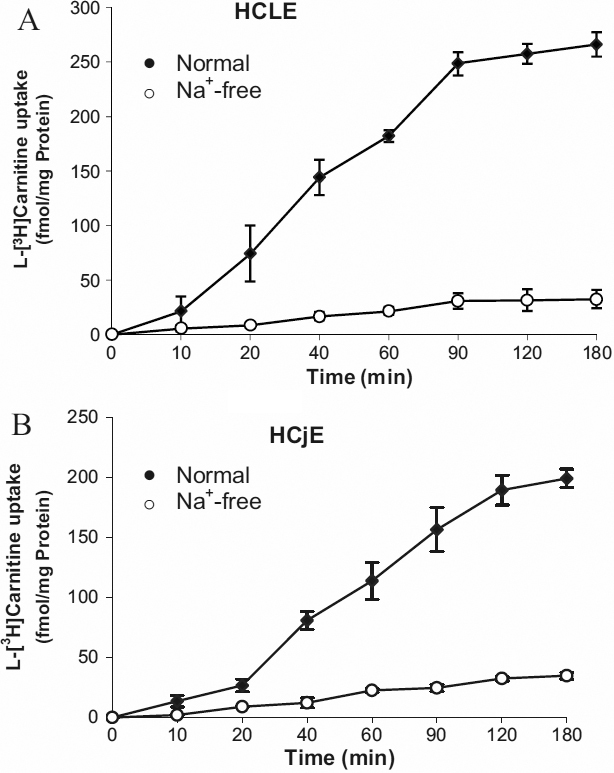
Time course and Na^+^-dependence of L-carnitine uptake. The uptake of 12nM L-[^3^H]carnitine by HCLE (**A**) or HCjE (**B**) cells was measured at pH 7.4 and 37 °C in the presence or absence of Na^+^. For Na^+^-free buffer, NaCl was replaced by an equimolar concentration of choline. Values are the mean±SD (n=4).

L-[^3^H] carnitine uptake in both HCLE (Figure 1A) and HCjE (Figure 1B) cells increased in a time-dependent manner, and appeared to be linear up to at least 90 min. When Na^+^ in the uptake buffer was replaced with choline, the uptake was decreased by 90% (p=0.001). Consequently, subsequent experiments were performed using an uptake period of 30 min in the presence of Na^+^.

### Kinetics of L-[^3^H]carnitine uptake in HCLE or HCjE cells

**Figure 2:**
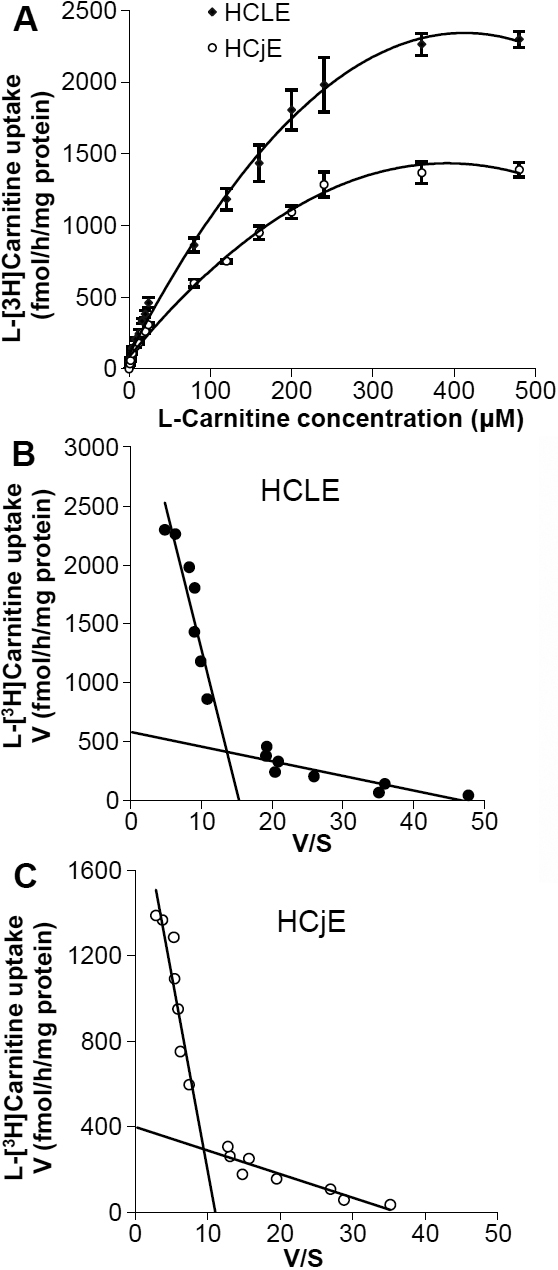
Concentration dependence and the kinetic characteristics of L-carnitine uptake.   **A**: Concentration dependence of carnitine uptake by HCLE or HCjE cells; **B **and** C: ** the kinetic characteristics of L-[^3^H]carnitine uptake with the Eadie–Hofstee plot indicating dependence of the uptake rate (*v*) on uptake rate/carnitine concentration (v/s) for HCLE (**B**) and HCjE (**C**) cells, respectively. HCLE or HCjE cells were pre-incubated for 60 min with uptake buffer, then different concentrations of L-carnitine were added to the incubation medium and the uptake was measured for 30 min at pH 7.4 and 37 °C in the presence of Na^+^. The concentration of L-[^3^H]carnitine was kept constant at 24 nM and the concentration of L-carnitine was varied (range 1-480 μM) by addition of unlabeled L-carnitine. Values are the mean±SD (n=4).

Carnitine uptake by HCLE or HCjE cells was concentration-dependent and the uptake appeared to be saturable (Figure 2A). The kinetics of L-[^3^H]carnitine uptake in HCLE and HCjE cells were analyzed by fitting the data to the Michaelis–Menten models using nonlinear regression analysis. The Eadie-Hofstee plot in which the uptake rate (v) was plotted as a function of the uptake rate/carnitine concentration ratio (v/s) clearly indicated two distinct components: a high-affinity and a low-affinity carnitine transport system in both HCLE (Figure 2B) and HCjE (Figure 2C) cells. The kinetic analysis yielded apparent Michaelis–Menten constants (*K*_m_) of 9.48±2.7 µM and 363.64±34.4 µM for high- and low-affinity carnitine transport, respectively, in HCLE cells (Table 2), and 9.39±1.3 µM and 196.03±17.1 µM for high- and low- affinity transport, respectively, in HCjE cells (Table 2). The maximum transport activities (*V*_max_) of 0.48±0.09 pmol/h/mg protein (high affinity) and 5.1±0.3 pmol/h/mg protein (low affinity) were estimated for HCLE, and 0.36±0.07 pmol/h/mg protein (high affinity) and 1.99±0.2 pmol/h/mg protein (low affinity) for HCjE (Table 2). These results suggest the existence of both high- and low- affinity L-carnitine transport systems in HCLE and HCjE cells.

**Table 2:** Determination of *K*_m_ and *V*_max_ of L-[^3^H]carnitine uptake in HCLE or HCjE cells at pH 7.4 and 37 °C.

** **	**HCLE**		**HCjE**	
**Parameters**	**High affinity**	**Low affinity**	**High affinity**	**Low affinity**
K_m_	9.48±2.7	363.64±34.4	9.39±1.3	196.03±17.1
V_max_	0.48±0.09	5.1±0.3	0.36±0.07	1.99±0.2
r^2^	0.9778	0.9862	0.9933	0.9796

Values are the mean±SD (n=3). K_m_=Michaelis constant (µM); V_max_=maximum initial transport activity (pmol/h/mg protein); and *r*^2^=goodness of fit (linear-factor).

### Energy-dependence of L-[^3^H]carnitine uptake by HCLE and HCjE cells

Following pretreatment with metabolic inhibitors sodium azide, dinitrophenol, or iodoacetic acid for 30 min, the uptake of L-[^3^H]carnitine was reduced to approximately 80%, 70%, and 30%, respectively (p<0.01), for both HCLE and HCjE cells (Table 3).

**Table 3:** Energy-dependence of the uptake of L-[^3^H]carnitine by HCLE or HCjE cells at pH 7.5 and 37 °C.

** **	**Relative uptake (% of control)**	
**Compound**	**HCLE**	**HCjE**
Sodium azide	80.4±1.8	79.2±1.3
Dinitrophenol	69.1±4.7	63.1±7.6
Iodoacetic acid	27.2±6.3	27.8±3.3

HCLE or HCjE cells were pre-incubated for 30 min in uptake buffer containing 10 mM of sodium azide, dinitrophenol or iodoacetic acid; L-[^3^H]carnitine (24 nM) was then added and the incubation continued for 30 min. Each value is the percentage uptake of L-[^3^H]carnitine in medium containing sodium azide, dinitrophenol, or iodoacetic acid compared with that in control medium without these compounds. Values are the mean±SD of results from four experiments.

### pH - dependence of the uptake of L-[^3^H]carnitine by HCLE and HCjE cells

**Figure 3:**
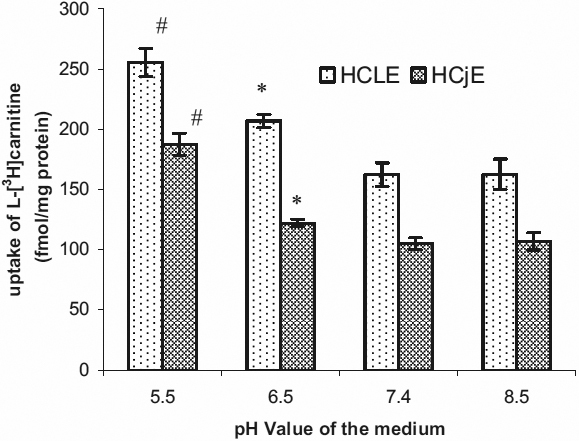
Effect of the pH of the medium on the uptake of L-[^3^H]carnitine by HCLE or HCjE cells at 37 °C. HCLE or HCjE cells were pre-incubated for 60 min in uptake buffer of different pH values at 37 °C; L-[^3^H]carnitine (24 nM) was then added and incubation was continued for 30 min. Each value is the mean±SD of results from three experiments. #p<0.005 for HCLE and 0.002 for HCjE cells respectively compared with medium at pH 7.4. *p=0.002 for HCLE and, p=0.007 for HCjE, respectively compared with medium at pH 7.4.

Figure 3 shows the uptake of 24 nM L-[^3^H]carnitine by HCLE or HCjE cells in the external uptake buffer at pH 5.5, 6.5, 7.4, or 8.5. The uptake activity was highest at pH 5.5 (p=0.005 and p=0.002 for HCLE and HCjE cells, respectively). The activity was higher at pH 6.5 than pH 7.4 (p=0.002 and p=0.007 for HCLE and HCjE cells, respectively), but no difference was found at pH 7.4 compared to pH 8.5. No changes in cell morphology were observed at pH 5.5 and pH 6.5 (data not shown).

### Structural analog, organic cation and BCH inhibition on the uptake of L-[^3^H]carnitine by HCLE and HCjE cells

As shown in Table 4, the structural analogs L-carnitine, D-carnitine, acetyl-L-carnitine and γ-butyrobetaine significantly inhibited the uptake of L-[^3^H]carnitine by both HCLE and HCjE cells. For both cell types, the inhibitory effect of L-carnitine was slightly greater than that of D-carnitine and acetyl-L-carnitine, and the effect of γ-butyrobetaine, a precursor of carnitine biosynthesis, was approximately equal to that of L-carnitine. No difference was found between D-carnitine and acetyl-L-carnitine. Inhibition was also concentration dependent. TEA, a known organic cation transported by OCTN2 [25], and BCH, a known specific inhibitor for ATB^0,+^ [22], both significantly inhibited L-[^3^H]carnitine uptake by HCLE and HCjE cells in a concentration dependent manner (Table 4).

**Table 4:** Inhibitory effect of structural analogs, organic cation and BCH on the uptake of L-[^3^H]carnitine by HCLE or HCjE cells.

** **	** **	**Relative uptake (% of control)**	
**Compound**	**Conc. (mM)**	**HCLE**	**HCjE**
L-Carnitine	0.5	34.6±2.4	24.9±2.1
** **	1	14.9±3.2	11.7±1.5
D-Carnitine	0.5	46.6±3.1	43.9±1.9
** **	1	14.4±0.6	21.7±1.1
Acetyl-L-Carnitine	0.5	42.1±4.8	37.1±4.9
** **	1	20.9±4.1	25.6±5.7
γ-Butyrobetaine	0.5	30.2±5.3	27.5±4.8
** **	1	16.9±3.7	17.4±3.3
TEA	0.1	85.6±4.2	74.9±2.7
** **	1	48.3±5.1	59.2±1.4
BCH	0.1	87.1±0.7	84.5±4.4
** **	10	46.5±2.9	63.8±4.3

HCLE or HCjE cells were pre-incubated for 60 min in uptake buffer at 37 °C; L-[^3^H]carnitine (24 nM) was then added in the absence (control) or presence of L-carnitine, D-carnitine, Acetyl-L-carnitine, γ-Butyrobetaine, TEA and BCH, and the incubation continued for 30 min. Data (uptake of L-[^3^H]carnitine as a percentage of that in the control) are means±SD of results from three experiments. All results were significantly reduced compared to the controls (p<0.01).

### mRNA expression of *ATB^0,+^*

**Figure 4:**
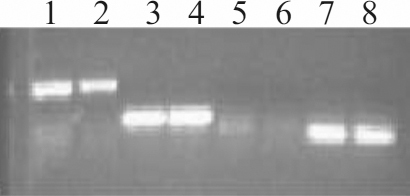
Relative expression of *OCTN1*, *OCTN2*, and *ATB^0,+^* in human ocular epithelial cells. Representative image of semi-quantitative RT PCR -amplified human *OCTN1*, *OCTN2*, *ATB^0,+^*, and *ACTB* products. In the image, Lanes 1,3,5,7 show HCLE product and Lanes 2,4,6,8 show HCjE product: Lanes 1–2 *OCNT1*; Lanes 3–4 *OCTN2*; Lanes 5–6 *ATB^0,+^*; and Lanes 7–8 *ACTB*.

The expression of *OCTN1* and *OCTN2* has been reported previously in HCLE and HCjE cells [10]. The finding in the present study where BCH was found to inhibit L-[^3^H]carnitine uptake, suggests that *ATB^0,+^* may be involved in L-carnitine transport in HCLE and HCjE cells. The expression of this transporter was detected but at a much lower level relative to *OCTN1* and *OCTN2* (Figure 4).

### The blocking effect of OCTN1 and OCTN2 antibody on the uptake of L-[^3^H]carnitine by HCLE and HCjE cells

**Figure 5:**
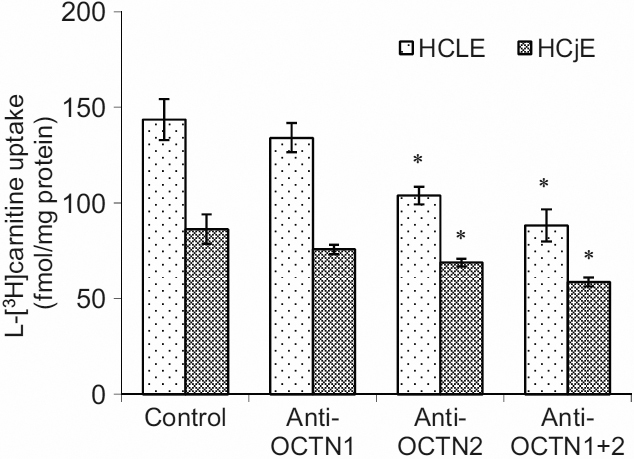
Blocking effect of OCTN1 and/or OCTN2 antibody on the uptake of L-[^3^H]carnitine by HCLE or HCjE cells at 37 °C. HCLE or HCjE cells were pre-incubated for 60 min in uptake buffer containing OCTN1 (1:500) and/or OCTN2 (1:500) antibody at 37 °C; L-[^3^H]carnitine (24 nM) was then added and incubation was continued for 30 min. Each value is the mean±SD of results from three experiments. *p<0.01 compared with the control group.

As shown in Figure 5, the anti-OCTN2 antibody blocked L-[^3^H]carnitine uptake in both HCLE (p=0.004) and HCjE (p=0.019) cells. The anti-OCTN1 antibody, on the other hand, did not significantly block uptake when compared with the control group nor did it contribute to blocking in combination with OCTN2 (Figure 5).

## Discussion

Carnitine transport has been extensively studied in a variety of human and animal tissues, such as kidney, skeletal muscle, heart, placenta, brain [20], mammary gland epithelia [11], liver [26], and rabbit conjunctiva [9]. However, little is known about the function of carnitine transporters present in ocular tissues including the corneal and conjunctival epithelia [10]. This present study provides further insights into the mechanism of carnitine transport in cultured human ocular epithelial cells, and the transporters specific for this process.

Our findings indicate that the uptake of L-[^3^H]carnitine in both HCLE and HCjE cells is saturable and Na^+^-dependent with the uptake profile for both cell lines comprising two distinct and significantly different components, suggesting the existence of both high and low affinity L-carnitine transport systems. The high affinity system *K*_m_, obtained in the present study for HCLE and HCjE cell lines (9.48 µM and 9.39 µM, respectively) is similar to that derived for the high affinity L-carnitine transporter, OCTN2, in various other tissues; for example, conditionally immortalized rat retinal capillary endothelial cells (29.0±13.8µM) [27], isolated rat kidney brush border membrane vesicles (17.4 µM) [28], LLC-PK1 cells (11.0 µM) [29], and CHO cells with functional expression of OCTN2 (8.01 µM) [30]. Our values are in good agreement with the range of 8.01–29 µM reported for the above studies performed at 37 °C, pH 7.4.

Metabolic inhibitors that uncouple oxidative phosphorylation (sodium azide and dinitrophenol) [26], as well as those that inhibit glycolytic ATP generation (iodoacetic acid) reduced the L-[^3^H]carnitine uptake, further indicating that L-carnitine is transported by an energy-dependent, active carrier-mediated transport system. The uptake activity was also found to be pH-dependent. Since changes in pH could affect the conformation of the transporter within the plasma membrane, this could have a direct effect on the transport of L-carnitine [26]. This pH-dependence might be ascribed to at least two underlying mechanisms, including activation by a proton gradient, or by the presence of a functionally optimal protonated form of the transporter [16]. The activity demonstrated here is similar to the activity of L-carnitine transport in the human placental brush-border membrane [15], but differs from that reported for cultured human hepatoma HLF cells, in which the uptake was highest at pH 7.4 [26]. This variation in optimal pH between different cell types warrants further investigation.

The involvement of the transporters, OCTN2 or ATB^0,+^, in the transport of carnitine was further confirmed by inhibition (substrate specific) studies. The concentration-dependent inhibition studies showed that carnitine transport activity was reduced by the carnitine analogs (L-carnitine, D-carnitine, acetyl-L-carnitine, and γ-butyrobetaine) and organic cationic compound (TEA). The inhibitory potencies of these compounds agree with those previously reported for the inhibition of OCTN2-mediated transport [31,32]. A recent study by Tachikawa and colleagues [33] found similar inhibition with L-carnitine and TEA in their studies across the inner blood-retinal barrier. They concluded that OCNT2 is most likely involved in L-carnitine transport. Similar inhibition was also observed in the OCTN2-transfected HEK cells [20]. Using polarized monolayers of HCLE and HCjE cells, we have previously demonstrated that the majority of carnitine was transported to the apical surface of the cells, consistent with the localization of OCTN2 found predominantly on apical membrane of the cells [10]. In agreement with the findings of others [20], our data here provide functional characteristic evidence of the OCTN2-actively mediated L-carnitine transport process for the human corneal and conjunctival epithelial cells. This may also offer further support for other reported findings where the uptake of L-carnitine into ocular tissues is observed [4,8,9].

ATB^0,+^, on the other hand, is a Na^+^- and Cl^-^- coupled transport system for neutral and cationic amino acids. It is a low-affinity transporter for L-carnitine and is sensitive to BCH at high concentrations (5–10 mM) [22]. In the present study, we also found that BCH (0.1 and 10 mM) inhibited the L-[^3^H]carnitine uptake in HCLE and HCjE cells with greater inhibition exhibited at higher concentrations. Further, the real-time PCR analysis detected a low level of ATB^0,+^ in these cells. These data suggest that ATB^0,+^ may also play a role in the L-carnitine transport process, as has been suggested previously [22,34]. In contrast to OCNT2, ATB^0, +^ does not transport acetyl-carnitine, highlighting differential affinities of OCTN2 and ATB^0,+^ which may dictate independent physiologic roles [22].

OCTN1 is a pH-dependent organic cation transporter that transports L-carnitine in a Na^+^-dependent manner [17,18]. Rat intestinal OCTN1, however, reportedly interacts with L-carnitine with low affinity and in a Na^+^-independent manner [35]. Conversely, OCTN2 transports carnitine with high affinity in a Na^+^-dependent manner and transports organic cations in a Na^+^-independent manner [15,19]. The observed blocking of L-[^3^H]carnitine uptake by OCTN2-specific antibodies, but not with OCTN1-specific antibodies, further supports the hypothesis that OCTN2 plays a major role in the transport of carnitine in human ocular epithelial cells and constitutes the high affinity transport component. The contribution of both OCTN1 and ATB^0,+^ need to be further addressed to elucidate the nature of the low affinity kinetic component.

L-carnitine is present in considerable quantities in the tears of normal healthy eyes [7]. However, for dry eye patients, the tear carnitine level is reduced significantly [7]. Corrales et al. [2] have shown that L-carnitine can protect against stress activation of corneal epithelial cells in response to hyperosmolar stress. We also demonstrated that carnitine protects corneal epithelial cells from hyperosmolar solution induced damage [3]. Taken together these observations support the hypothesis that carnitine plays a crucial role in protecting ocular surfaces from hyperosmolarity-induced damage, thus contributing to the reduction of deleterious physiologic changes in dry eye. In addition, this present work provided evidence that carnitine can be actively transported to the ocular cells in a Na^+^-dependent manner, potentially extending the application of carnitine not only as a micronutrient but also as a beneficial compatible solute in topically administered ophthalmic formulations, such as artificial tears, to enhance cell survival under hypertonic conditions.

In conclusion, high- and low-affinity L-carnitine transport occurs in human HCLE and HCjE cells. Consistent with our previous demonstration of OCTN2 expression in these cells, the present work provides further evidence of an active high-affinity transport system for carnitine in the ocular epithelial cells that is Na^+^-, pH-, and energy-dependent with characteristics resembling OCTN2.
